# Diabetes Patients' Experiences With the Implementation of Insulin Therapy and Their Perceptions of Computer-Assisted Self-Management Systems for Insulin Therapy

**DOI:** 10.2196/jmir.3198

**Published:** 2014-10-23

**Authors:** Airin CR Simon, Wouter T Gude, Frits Holleman, Joost BL Hoekstra, Niels Peek

**Affiliations:** ^1^University of Amsterdam/Academic Medical CenterInternal Medicine/Medical InformaticsAmsterdamNetherlands; ^2^Academic Medical CenterMedical InformaticsUniversity of AmsterdamAmsterdamNetherlands; ^3^Academic Medical CenterInternal MedicineUniversity of AmsterdamAmsterdamNetherlands; ^4^Health e-Research CentreThe University of ManchesterManchesterUnited Kingdom

**Keywords:** Type 2 diabetes mellitus, clinical decision support systems, patient acceptance of health care

## Abstract

**Background:**

Computer-assisted decision support is an emerging modality to assist patients with type 2 diabetes mellitus (T2DM) in insulin self-titration (ie, self-adjusting insulin dose according to daily blood glucose levels). Computer-assisted insulin self-titration systems mainly focus on helping patients overcome barriers related to the cognitive components of insulin titration. Yet other (eg, psychological or physical) barriers could still impede effective use of such systems.

**Objective:**

Our primary aim was to identify experiences with and barriers to self-monitoring of blood glucose, insulin injection, and insulin titration among patients with T2DM. Our research team developed a computer-assisted insulin self-titration system, called PANDIT. The secondary aim of this study was to evaluate patients’ perceptions of computer-assisted insulin self-titration. We included patients who used PANDIT in a 4-week pilot study as well as patients who had never used such a system.

**Methods:**

In-depth, semi-structured interviews were conducted individually with patients on insulin therapy who were randomly recruited from a university hospital and surrounding general practices in the Netherlands. The interviews were transcribed verbatim and analyzed qualitatively. To classify the textual remarks, we created a codebook during the analysis, in a bottom-up and iterative fashion. To support examination of the final coded data, we used three theories from the field of health psychology and the integrated model of user satisfaction and technology acceptance by Wixom and Todd.

**Results:**

When starting insulin therapy, some patients feared a lifelong commitment to insulin therapy and disease progression. Also, many barriers arose when implementing insulin therapy (eg, some patients were embarrassed to inject insulin in public). Furthermore, patients had difficulties increasing the insulin dose because they fear hypoglycemia, they associate higher insulin doses with disease progression, and some were ignorant of treatment targets. Patients who never used a computer-assisted insulin self-titration system felt they had enough knowledge to know when their insulin should be adjusted, but still believed that the system advice would be useful to confirm their reasoning. Furthermore, the time and effort saved with automated insulin advice was considered an advantage. Patients who had used PANDIT found the system useful if their glycemic regulation improved. Nevertheless, for some patients, the absence of personal contact with their caregiver was a drawback. While guidelines state that adjustment of basal insulin dose based on fasting plasma glucose values is sufficient, both patients who had and those who had not used PANDIT felt that such a system should take more patient data into consideration, such as lifestyle and diet factors.

**Conclusions:**

Patients encounter multiple obstacles when implementing insulin therapy. Computer-assisted insulin self-titration can increase patient awareness of treatment targets and increase their confidence in self-adjusting the insulin dose. Nevertheless, some barriers may still exist when using computer-assisted titration systems and these systems could also introduce new barriers.

## Introduction

The rising incidence of type 2 diabetes mellitus (T2DM) is a major public health concern and financial burden [1]. Complications associated with this disease can be reduced by lowering blood glucose levels to near normal values with diet, oral glucose lowering treatment, and/or insulin therapy. For many patients, diet and oral glucose lowering treatment are insufficient to maintain good glycemic control in the long term. The progressive deterioration of the beta-cell function necessitates the addition of insulin to their existing treatment. Many clinical trials have shown that good glycemic control can be achieved in the majority of patients on insulin therapy [2]. International guidelines recommend a glycemic target of HbA1c (glycated hemoglobin) <7% (53 mmol/mol) to reduce diabetes-related complications [3,4]. In the United Kingdom and in the United States, only 59% and 47% of patients with T2DM achieve HbA1c values of 7.4% and 6-8% [5,6]. The glycemic target is also not achieved in most patients with T2DM in the Netherlands [7].

Insulin therapy requires frequent self-monitoring of blood glucose (SMBG), that is, observing and recording daily blood glucose levels over time to facilitate accordant adjustment of the insulin dose, called insulin titration [8]. While SMBG and injection of insulin are performed mostly at the patients’ homes [9], the majority of patients with T2DM requiring insulin therapy have their doses titrated by their care providers [10]. Failures to reach treatment target in clinical practice are partly due to the fact that patients have difficulties implementing insulin therapy; a nationwide study in the United States showed that 29% of patients with T2DM treated with insulin never practiced SMBG [11]. A systematic review published in 2004 reported that adherence rates to insulin varied from 62% to 64% in patients with T2DM in developed countries [12]. Furthermore, evidence suggests that patients remain on low doses of insulin and that their insulin doses are adjusted insufficiently to achieve treatment targets [3].

Several studies have shown that self-management is a key component of effective care for chronic diseases, such as T2DM, leading to improved patient outcomes [13]. If insulin titration can also be undertaken successfully by patients with T2DM themselves, this might improve glycemic control. Among patients with type 1 diabetes mellitus (T1DM), insulin self-titration is already well-established suggesting that therapeutic self-management may be similarly beneficial when applied to patients with T2DM [14].

Computer-assisted decision support is an emerging modality to assist patients with chronic diseases in general and patients with diabetes in the self-management of their disease [15]. Many existing systems in this area focus on insulin self-titration to support patients with T1DM in calculating the optimal pre-meal short-acting insulin dose [16]. Patients with T2DM who become insulin-dependent start with a basal insulin and thus require a different titration strategy [17]. To date, the majority of systems for patients with T2DM allow patients to enter blood glucose values in an electronic diary and are complemented by telemedicine functionalities to receive recommendations from medical professionals after the glucose readings are assessed [18,19]. More advanced systems automatically provide insulin dosing advice through decision support technologies [20]. While such computer-assisted insulin self-titration systems focus mainly on helping patients overcome barriers to the cognitive components of insulin titration, there may be other barriers, such as psychological or physical barriers, that still impede effective use of such systems. Furthermore, the use of computer-assisted systems by patients could induce new barriers during their self-management.

Many studies have focused on caregivers’ and patients’ resistance to the initiation of insulin therapy [21-23]. Studies have also identified barriers to the evaluation of blood glucose levels [24-27] and to insulin injection [12,28,29]. Yet, to our knowledge, no study has focused on patients’ experiences with and barriers to insulin titration. Also, no study has examined patients’ perceptions of computer-assisted insulin self-titration, investigating the feasibility of these decision support systems to be implemented in the near future. Our research group developed a computer-assisted insulin self-titration system for patients using once daily basal insulin, called the Patient Assisting Net-based Diabetes Insulin Titration (PANDIT) system [30]. In this qualitative study, our primary aim was therefore to identify experiences with and barriers to SMBG, insulin injection, and insulin titration among patients with T2DM using in-depth, semi-structured interviews. These experiences and barriers to insulin self-management were analyzed using three theories from the field of health psychology. The secondary aim of this study was to evaluate the perceptions of patients who used PANDIT in a pilot study as well as patients who never used such a system. This second analysis was based on the integrated model of user satisfaction and technology acceptance by Wixom and Todd [31].

## Methods

###  Study Participants

Patients with T2DM, aged 18-80 years, who were on insulin therapy, were considered eligible to participate in this study, irrespective of their glycemic control. In this study, we aimed to include 10 participants who had never used a computer-assisted insulin self-titration system (Group 1). Furthermore, 10 patients who were enrolled in a 4-week pilot involving the use of PANDIT were asked to participate in this study (Group 2). All participants were randomly recruited from a 1000-bed university hospital in Amsterdam, and general practices in and around Amsterdam, the Netherlands.

### Description of PANDIT

PANDIT is a Web-based system that provides insulin dosing advice for patients using once daily basal insulin. Exogenous basal insulin complements the residual insulin that is supplied by the pancreas. Basal insulin doses are traditionally titrated according to fasting plasma glucose (FPG) values [32].

**Figure 1:**
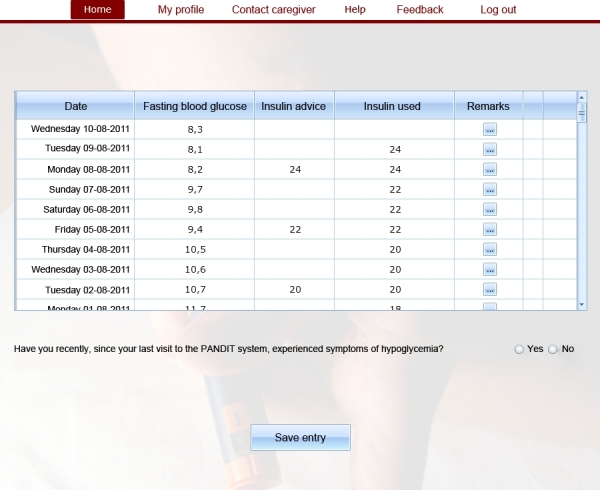
Screenshot of PANDIT.

The patient user interface of PANDIT resembles a glucose diary in order to facilitate the collection of FPG values. The patient user interface of PANDIT is displayed in Figure 1. After a patient has logged in and opened the diary, a one-page screen is displayed containing five columns that show calendar date, FPG values, insulin dosing advice as provided by the system, current dose of insulin used, and remarks. Patients need to access PANDIT at least once every 3 days to enter recently measured FPG values and their current insulin dose. Furthermore, they have to indicate whether they have experienced symptoms of hypoglycemic episodes.

A decision support algorithm uses the measured FPG values to determine whether the patient’s FPG is within the target range. If this is the case, the patient is advised to continue the current insulin dose, otherwise, the system will advise to adjust the insulin dose. If there is a reason for more intensively guided treatment, for example, if the patient frequently experiences hypoglycemic episodes, PANDIT will be automatically blocked from generating new insulin dosing advice and the care provider receives a message from PANDIT.

In addition to the decision support algorithm, PANDIT also incorporates asynchronous telemedicine functionalities. The telemedicine functionalities allow care providers to provide insulin dosing advice through the PANDIT interface to their patients. These telemedicine functionalities are automatically triggered when the system is blocked from generating advice as described above, but can also be evoked by care providers when they think this is necessary. As soon as the patient is sufficiently stabilized, the care provider can decide to “unblock” the algorithm, and let PANDIT generate new insulin dosing advice again.

###  Data Collection

In-depth, semi-structured interviews were conducted individually with all participants of the study. One researcher (ACRS) conducted face-to-face interviews with the participants of Group 1 to investigate their experiences with and barriers to SMBG, insulin injection, and insulin titration, as well as their perceptions of computer-assisted insulin self-titration. The face-to-face interviews were conducted at the outpatient clinic. Another researcher (WTG) conducted telephone interviews with the participants of Group 2 specifically focusing on their experiences with PANDIT. Face-to-face interviews had an average duration of 60 minutes. Telephone interviews lasted around 20 minutes. All interviews were audio-taped with oral consent of the participants. Table 1 lists the topics that were addressed during the interviews. Open-ended questions with neutral probes related to each topic were posed and follow-up questions were used to elicit more in-depth information.

**Table 1:** List of topics that were addressed during the interviews.

Topic			Item
**Face-to-face interviews (Group 1)**			
	**A. Experiences with and barriers to insulin titration**		
		To what extent patients adhere to…(a-c)	(a) performing SMBG^a^
		How patients feel about…(a-c)	(b) injection of insulin
		The ideal conditions to…(a-c)	(c) titration of insulin
		Difficult conditions to…(a-c)	
	**B. Perceptions of computer-assisted insulin self-titration**		
		To what extent patients are skillful in using…(a,b)	(a) computer
		How patients feel about using…(a-c)	(b) telemedicine
			(c) computer-assisted insulin self-titration system
**Telephone interviews (Group 2)**			
	**Experiences with and barriers to the use of PANDIT** ^b^		
		Positive or negative aspects/functionalities of PANDIT	
		Missing aspects/functionalities of PANDIT	
		Feelings about the insulin dosing advice given through PANDIT	

^a^SMBG: self-monitoring of blood glucose

^b^PANDIT: Patient Assisting Net-based Diabetes Insulin Titration system

### Data Analysis

The interviews were transcribed verbatim and analyzed qualitatively. In the analysis, we used the MAXQDA software [33]. For the primary aim, two researchers (ACRS and WTG) individually extracted all remarks regarding patients’ experiences with and barriers to SMBG, insulin injection, and insulin titration from the face-to-face interviews with Group 1. For the secondary aim, the two researchers extracted the perceptions of computer-assisted insulin self-titration addressed by participants in Group 1 and the experiences with PANDIT addressed by participants in Group 2.

### Analysis of Experiences With and Barriers to Insulin Self-Management

The analysis of the interview data was based on the steps described in the handbook of Green et al [34]. To classify the textual remarks, we created a codebook during the analysis, in a bottom-up and iterative fashion, following the procedure for codebook creation described by DeCuir-Gunby et al [35]. The codes were developed from the data rather than from existing theories, and we examined the data multiple times and revised the codebook and the assigned codes if necessary, until there were no more changes either to the codebook or the assigned codes. During the second and later cycles of examination, we used constructs from three selected theories from the field of health psychology (Stages of Change Model [36], Theory of Planned Behavior [37], and Self-Regulatory Theory [38]) to guide changes to the codebook. However, data was never “forced” into these theories. If no suitable construct was found, the original code that was derived from the data was retained.

**Figure 2:**
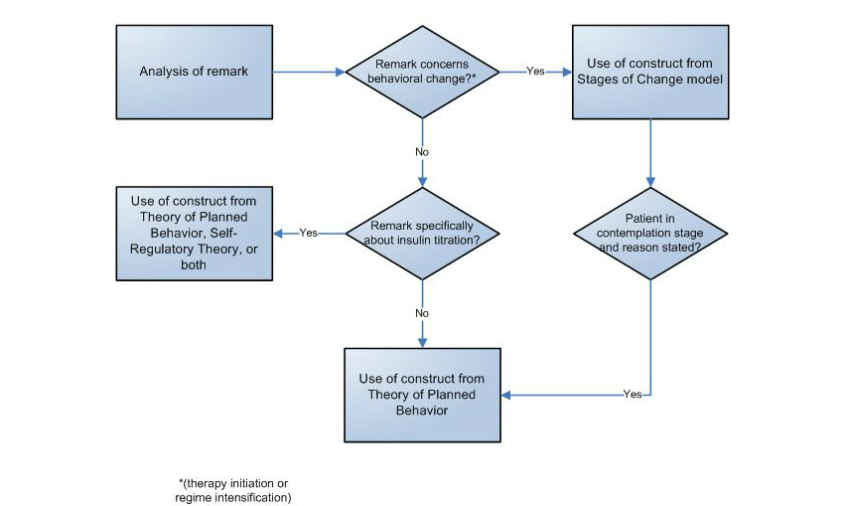
Flowchart of matching bottom-up coded remarks to constructs from theoretical frameworks.

Figure 2 depicts the process that was followed to match bottom-up coded remarks to constructs from the three theories from health psychology. The Stages of Change Model of Prochaska and DiClemente distinguishes five stages on the way to behavioral change, namely precontemplation, contemplation, preparation, action, and maintenance [24]. The Stages of Change Model hence was used to appreciate patients’ readiness to perform insulin self-management, requiring acknowledgement of starting insulin therapy or an additional insulin injection (insulin regime intensification). Among patients situated in the action stage of the Stages of Change Model, Ajzen’s Theory of Planned Behavior was used to explore participants’ attitudes toward a behavior, their perceived behavioral control, and subjective norms as factors that influence their behavioral intention [37]. Specifically for experiences with and barriers to the task of insulin titration, Leventhal et al’s Self-Regulatory Theory was used to explore illness representations based on perceptions of cause, identity, timeline, consequences, and controllability of diabetes in terms of prevention and cure [38]. Table 2 gives an overview of all theoretical constructs that were used during data analysis. The two coders (ACRS, WTG) resolved discrepancies through consensus. In case of disagreement, a third researcher (NP) was consulted. Having a third party examine the coding choices made by team members is a technique that has also been used in previous qualitative studies [39]. The entire final codebook is listed in Multimedia Appendix 1.

Two researchers (ACRS and WTG) independently categorized the remarks according to three different topic schemes: (1) the medical task addressed (SMBG, insulin injection, or insulin titration), (2) whether the remark concerned a (perceived) barrier or an experience, and (3) the construct from one of the theories. When participants reported that an action was not performed, not performed in a timely fashion, or not performed correctly, the reported cause of this circumstance was classified as a barrier to performing the action. We distinguished two types of barriers: barriers that were reported by participants themselves, and barriers that were induced from participants’ remarks by the researchers. To this end, an experienced diabetes physician (ACRS) verified whether patient reported behavior was in line with common recommendations in the field of diabetes, and if this was not the case, the cause of this circumstance was classified as an induced barrier. For example, if patients reported that they did not see advantages of glucose-lowering actions for blood glucose levels that were evidently too high, this was classified as an induced barrier caused by a lack of knowledge of the long-term risks of diabetes or of treatment targets. All patient experiences were classified as “positive” or “negative”, depending on the feelings reported by the participant in question.

**Table 2:** Overview of theoretical constructs used in data analysis.

Theory	Focus	Constructs		Description^a^
Stages of Change Model [36]	Individual’s motivation and readiness to change a behavior	Stages	Precontemplation	Not yet acknowledging that there is a problem behavior that needs to be changed
			Contemplation	Acknowledging that there is a problem but not yet ready or sure of wanting to make a change
			Preparation	Getting ready to change
			Action	Changing behavior
			Maintenance	Maintaining the behavior change
Self-Regulatory Theory [38]	Individual’s cognitive representations of their current health status based on illness representation	Illness representation	Cause of illness	Perceived causes of an illness
			Identity with the illness	Name or label of an illness
			Consequences	Anticipated and experienced consequences of an illness
			Timeline	Perception of progress and duration of an illness
			Controllability	Perception of amenability to control or cure an illness
Theory of Planned Behavior [37]	Relations between an individual's beliefs, attitudes, intentions, behavior and perceived control over that behavior	Factors	Attitude toward behavior	Positive or negative feelings about performing a behavior
			Subjective norm	Perception of whether people important to the individual think the behavior should be performed
			Perceived behavioral control	Perception of the difficulty of performing a behavior
Integrated model of User Satisfaction and Technology Acceptance [31]	Causal chain from key characteristics of system design to beliefs and expectations about outcomes that ultimately determine usage	Factors	Completeness	Degree to which the system provides all necessary information
			Accuracy	Perception that the information is correct
			Format	Perception of how well the information is presented
			Currency	Perception of the degree to which the information is up to date
			Reliability	Dependability of system operation
			Flexibility	Way the system adapts to changing demands of the patient
			Integration	Way the system allows data to be integrated from various sources
			Accessibility	Ease with which information can be accessed from the system
			Timeliness	Degree to which the system offers timely responses to requests for information or action

^a^In the context of our study, the term “behavior” in this column can be interpreted as either (1) performing self-monitoring of blood glucose, (2) injecting insulin, or (3) titrating insulin.

### Analysis of Perceptions of and Experiences With Computer-Assisted Insulin Self-Titration

The perceptions of computer-assisted insulin self-titration that emerged from the face-to-face and telephone interviews were similarly coded bottom-up to eventually develop a codebook. Here too, if consensus could not be reached, a third researcher (NP) was consulted. To support examination of the final coded data of Group 2, two researchers matched each code to a construct from the integrated model of technology acceptance and user satisfaction constructed by Wixom and Todd [31]. This model provides a mechanism for understanding how system and information characteristics influence intended usage. It proposes that system characteristics (reliability, flexibility, integration, accessibility, and timeliness) and information characteristics (completeness, accuracy, format, and currency) influence system and information quality, respectively. Following a causal chain, system and information quality influence object-based attitudes toward the system that can shape behavioral beliefs of usefulness, ease of use, and, ultimately, system usage. For example, if a computer system is hard to access, this will negatively influence its perceived usefulness and users may decide to use other systems of information sources instead. Table 2 (bottom row) lists the theoretical constructs from this model. The final coded data of Group 1 were not matched to constructs of a model as these patients were never presented with the computer-assisted insulin self-titration system.

## Results

### Participants

To recruit the 10 participants who had never used a computer-assisted insulin self-titration system, 12 patients were invited. All 10 patients who participated in the pilot study with PANDIT agreed to take part in this study. Participant characteristics are displayed in Table 3. In the following sections, we first describe the experiences with and barriers to insulin self-management categorized by medical task. Subsequently, we describe patients’ perceptions of, and experiences with, computer-assisted insulin self-titration per patient group.

**Table 3:** Baseline characteristics of study participants by group.

Characteristic			Group 1 (n=10)	Group 2 (n=10)
**Demographic characteristics**				
	Age (years), mean (SD)		53.9 (7.7)	56.9 (8.9)
	Male sex, n (%)		6 (60%)	6 (60%)
	**Education level** ^a^ **, n (%)**			
		Low education level	3 (30%)	2 (20%)
		Middle education level	4 (40%)	4 (40%)
		High education level	3 (30%)	4 (40%)
**Diabetes characteristics**				
	Diabetes duration since diagnosis (years), median (min-max)		8.0 (2.0-25.0)	7.0 (1.0-23.0)
	Duration of insulin use (years), median (min-max)		3.0 (0.3-15.0)	1.0 (0.0-3.0)
**Insulin regimen**				
	Basal, n (%)		7 (70%)	10 (100%)
	Multiple daily, n (%)		3 (30%)	0 (0%)
	HbA1c, mean (SD)		7.2 (0.7)	8.4 (6.3)

^a^Low education level: primary school or none; Middle education level: vocational or other secondary school; High education level: university or vocational postsecondary school.

### Experiences With and Barriers to Insulin Self-Management

In total, the face-to-face interviews yielded 77 unique remarks regarding experiences with and barriers to insulin self-management (Multimedia Appendix 1). A total of 25 remarks were categorized as barriers, of which six were induced by the diabetes physician. Most barriers concerned the task of insulin titration. For three remarks, no suitable construct was found, for which we added one new construct: “fit between medical commitment and daily routine”, implicating the impact of a patient’s daily activities on the performance of a medical task. To define patients’ attitudes more specifically, we added three child-constructs under the construct “attitude towards behavior” (Theory of Planned Behavior), namely “perceived usefulness”, “physical impact”, and “effect on daily life”. We also added one child-construct under the construct “perceived behavior control” (Theory of Planned Behavior), namely “perceived cognitive load”, implicating the emotional or cognitive burden of the medical task. No remarks concerned the constructs “cause of illness” or “identity with the illness” (Self-Regulatory Theory), or “preparation” (Stages of Change Model).

### Insulin Therapy Initiation and Insulin Regime Intensification

Five remarks were made about insulin therapy initiation or insulin regime intensification (ie, adding a bolus insulin before a meal). Most patients reported having a reluctance to using an injectable drug due to fear of a lifelong commitment to insulin therapy or fear of disease progression (“contemplation stage”, Stages of Change Model).

I just can’t stand the injection. If it would be once a day, it would be fine. But I had to inject twice a day. …Two times is really the maximum for me. They proposed to me to inject three times, but I said no. I don’t want that. I couldn’t do it.

Female, age 46, Group 1, participant 7

There were also patients who experienced positively that initiating insulin therapy was effective in lowering blood glucose values.

### Self-Monitoring of Blood Glucose

A total of 13 remarks were made about SMBG. Many patients reported that the performance of measurement of blood glucose was accompanied by feelings of pain or tenderness in the fingertips (“physical impact”, child construct of “attitude towards behavior”, Theory of Planned Behavior). However, these patients reported that, despite the pain, they still performed the measurements.

I still think finger pricks are a disaster. It just hurts.…I have tried different lancets but all of them hurt. …OK, I have to, I have to, but still I don’t like those horrible nasty pricks.

Female, age 60, Group 1, participant 1

Some patients indicated that the painful feelings were outweighed by the usefulness of SMBG to detect aberrant blood glucose values and to become acquainted with the effects of daily activities and insulin on blood glucose values.

I used to measure 4-6 times a day. At a certain point you get acquainted with hypoglycemia and learn to sense a hypoglycemic event coming up. …After a year I knew how much I food had to eat and insulin I had to inject to prevent a hypoglycemic event.

Male, age 40, Group 1, participant 4

However, these patients no longer perceived these frequent SMBG as useful when their blood glucose became better controlled and thus were mostly within the target range.

### Insulin Injection

A total of 31 remarks were made about insulin injection. Many patients on insulin therapy mentioned injection pain and site reactions localized on the skin, such as bruises and swellings, as a drawback of insulin therapy (“physical impact”, child construct “attitude towards behavior”, Theory of Planned Behavior). Nevertheless, the physical impact of insulin injections did not necessarily withhold patients from compliant use of insulin therapy.

I inject the rapid-acting insulin in my abdomen and I don’t like it. I also have bruises on my abdomen and it’s more painful and more sensitive. …This wouldn’t stop me from injecting, but I do avoid the injection site for some days to give it time to recover.

Female, age 59, Group 1, participant 3

Another negative feeling toward insulin injection that many patients elaborated on was that the commitment to insulin therapy negatively affected their daily personal or social life (“effect on daily life”, child construct “attitude towards behavior”, Theory of Planned Behavior).

If someone asks: do you want to join us? …I can’t, because I did not bring a sandwich …Or if someone proposes at 5 PM to go to the café and have dinner, I also can’t because I did not bring my injection kit. I always have to carefully consider bringing something or not.

Female, age 60, Group 1, participant 1

Also, several patients mentioned they felt ashamed to inject their insulin in public. Whereas most sought privacy in the restroom to attend their medical needs, others avoided social occasions (“subjective norm”, Theory of Planned Behavior).

If I’m with other people, I dislike injecting. In my personal environment, my wife is the only one who knows [of my disease]. …I don’t feel the necessity to tell others. If I have to inject in public, I retreat. I have to say I avoid this kind of situation. I don’t often go to family parties and such things.

Male, age 40, Group 1, participant 4

Rapid-acting insulin should be administered just before a meal, except when there is a medical reason (eg, delayed gastric emptying) not to do so. However, some participants reported deviating from this advice. One found it intuitively more logical to inject insulin after the meal (“attitude towards behavior”, Theory of Planned Behavior). Another patient initially injected his insulin before a meal but once experienced a hypoglycemic event when dinner in a restaurant was served later than expected and he had already injected the insulin. Hereafter, he switched to injecting his insulin after the meal. Difficulties with estimating how much one would eat were also named as a reason for deviation (“perceived behavioral control”, Theory of Planned Behavior).

### Insulin Titration

A total of 30 remarks were made about insulin titration. In particular, increasing the insulin dose was considered a drawback. Patients said that in the short term, increasing the insulin dose led to a fear of developing hypoglycemia (“consequences”, Self-Regulatory Theory). In the long term, they feared that increasing the insulin dose would lead to disease progression or a transition to a multiple daily insulin regimen (“timeline”, Self-Regulatory Theory).

They said increase [your insulin dose] to 18 [insulin units], increase to 24. Then I thought: it’s getting worse. Am I getting sicker? …I didn’t know this was normal in the initial phase. …It takes some time before you realize how it works.

Male, age 54, Group 1, participant 2

One patient incorrectly perceived that high doses of insulin might interact with other medication (“perceived behavioral control”, Theory of Planned Behavior). Another patient also indicated being reluctant to increase the insulin dose as this would reduce insight in the remaining function of the pancreas (“controllability”, Self-Regulatory Theory).

If you inject more insulin if your blood sugar is too high, you end up in a situation where you’re dependent on [exogenous] insulin. Say you have a steady lifestyle and a fixed insulin dose. …If your [blood glucose] values decrease, it could mean that the Islets of Langerhans function better. If you constantly switch insulin doses, you can’t see if you’re doing better.

Male, age 40, Group 1, participant 9

With regard to self-adjustment of insulin dose, many patients reported that they found it difficult to choose a correct insulin dose. Some patients were not aware of correct glucose targets. Consequently, patients continued their insulin dose when they actually should have increased it, or even decreased their insulin dose when they actually should have continued it.

If my blood glucose value is near 10 or 10.2 [mmol/L], I’m not very alarmed. …If it would be near 15 [mmol/L], I could call [my diabetes nurse]. …Until 10 point something it does not bother me.

Male, age 54, Group 1, participant 2

Also, some patients felt that they were unable to control their blood glucose values due to the large number of external, disturbing factors such as irregular working hours, poor sleeping, and influenza episodes. They would typically consider the evolution of their blood glucose values as unpredictable and varying for no apparent reason (“controllability”, Self-Regulatory Theory).

### Other Barriers

Patients indicated that the obligation to use insulin on top of other therapies increased the mental burden of disease management (“perceived cognitive load”, child construct of “perceived behavior control”, Theory of Planned Behavior). Therefore, these patients preferred to limit the frequency of performing any medical procedure to a minimum.

I prefer to perform as few medical procedures as possible. I already take medication because my thyroid was removed, that’s why I’ve become overweight and that’s how I got diabetes. Therefore, I try to limit everything to a minimum. Everybody prefers to be healthy and without needs.

Female, age 59, Group 1, participant 3

Furthermore, daily activities and irregular working hours were named as barriers to integrate SMBG and insulin injection in their daily routine (“fit between medical commitment and daily routine”, added construct). As a result, these tasks were often forgotten or delayed.

### Perceptions of and Experiences With Computer-Assisted Insulin Self-Titration

The telephone interviews and face-to-face interviews yielded a total of 20 and 18 remarks respectively regarding perceptions of computer-assisted insulin self-titration. The final coded data of Group 1 were not matched to constructs of a model. These patients mainly discussed whether they found it useful and/or efficient and whether they could trust the system. With regard to Group 2, remarks regarding functionalities that were missing in PANDIT were assigned to an added construct to the Theoretical Integration of User Satisfaction and Technology Acceptance: “missing functionalities”. Furthermore, for three remarks, we added two other constructs*:* “perceived usefulness”, implicating the degree to which a person believes that his or her diabetes would benefit from the use of a computer-assisted insulin self-titration system; and “attitude towards behavior”, implicating the positive or negative feelings about performing computer-assisted insulin self-titration. The constructs “accuracy”, “currency”, “reliability”, “integration”, and “timeliness” were not applicable.

Patients who had never used a computer-assisted insulin-titration system (Group 1) and who expected a computer-assisted insulin self-titration system to be useful felt they had enough knowledge to know when their insulin should be adjusted, but still believed that the system advice would be useful as it could confirm their reasoning.

When patients were asked how they would feel about receiving insulin dosing advice at home, almost all appreciated that it could save time and effort in comparison to face-to-face contacts and telephone calls with their caregiver.

It could save me time. Sometimes you have to wait here [in the hospital] for quite a while. …Other times there are telephone appointments but that also requires waiting if the line is busy. The use of such a system would save both parties time. If it’s easy and possible [to titrate the insulin dose] without speaking to the person I would like that, yes.

Male, age 54, Group 1, participant 2

The main reason for patients who had used PANDIT in a pilot study (Group 2) to find PANDIT useful was that using the system resulted in an evident improvement of their blood glucose levels (“perceived usefulness”*,* added construct).

Now that I measure my fasting blood glucose values [and enter my fasting blood glucose values in the system], my daily sugar value is around 6 whereas it used to be over 10 [mmol/L]! I’m very happy with the system. …I only feel better.

Female, age 47, Group 2, participant 11

Furthermore, some of these patients generally appreciated the ease with which information could be accessed from the system and the fact that professional guidance was offered through the system when their medical situation had changed (“flexibility”).

If I have a pain attack or the flu everything gets disrupted. If I let them [the caregivers] know how the high or low [blood glucose] value occurred, they can intervene if necessary.

Male, age 64, Group 2, participant 18

Patients of Group 1 who saw no added value of using such a system typically felt confident to perform insulin titration themselves, or were content with current diabetes management practice.

Why would I want to regulate my [basal] insulin? …I don’t think it’s useful for me …perhaps [it would be useful for] people who have difficulties with their diabetes and don’t have it for as long as I do.

Female, age 60, Group 1, participant 1

Some patients of Group 1 expressed their difficulties in trusting insulin dosing advice provided by a computer system, in particular if insulin dosing advice was solely based on blood glucose values. Likewise, patients who had used PANDIT in a pilot study (Group 2) suggested that the system should also take into account other factors that could influence blood glucose level, such as diet and lifestyle (“completeness”).

I think [computer-assisted insulin self-titration] might not be realizable, because the system will not know your exercise rhythm. If I go fishing one day, then I know what insulin dose I’m going to administer. But if I suddenly give up fishing and go for a 90-minute run, I have already entered my data, and they [caregivers] will not know that I’m exercising.

Male, age 56, Group 1, participant 5

Currently it's [the determination of new insulin doses] only focused on the glucose values, but of course other influences are nutrition and lifestyle. Would it be useful to extend the system with information on the lifestyle and food intake?

male, age 64, Group 2, participant 18

Furthermore, patients of Group 2 sometimes missed personal contact when advice was provided.

I miss the personal contact with the diabetes nurse. …In August, I started with insulin and every 2 weeks I called my diabetes nurse. I had personal contact by telephone, and even came to the hospital a couple of times, and now things go through email. I find contact by telephone more enjoyable than by computer.

female, age 68, Group 2 participant 16

## Discussion

### Principal Results

In this study, we identified experiences with and barriers to SMBG, insulin injection, and insulin titration. Furthermore, we explored perceptions of computer-assisted insulin self-titration among patients with T2DM. Some patients who considered starting with insulin therapy feared a lifelong commitment to insulin therapy, disease progression, or had a general reluctance to using an injectable drug. Once on insulin therapy, patients generally experienced it as being effective. Yet, many barriers can arise when performing insulin injection and insulin titration. Insulin injection is painful, a burden to daily life, and some patients considered it embarrassing to inject in public. Also, some patients administered their rapid-acting insulin incorrectly, namely after mealtime instead of before. This behavior was considered more intuitive and provided more control as it is difficult to predict how much one will eat. With regard to insulin titration, most patients found it difficult to increase the insulin dose. This was caused by a perceived increased risk of hypoglycemia, fear of disease progression, and a perceived lack of control, due to the apprehension that increasing the dose might cause problems. Also, some patients did not know how to adjust their insulin, due to ignorance of treatment targets and unpredictable blood glucose levels. Measurement of blood glucose was considered painful, but this did not withhold them from doing it. Furthermore, insulin therapy was found difficult to integrate with other therapies, and difficult to integrate with daily activities or irregular working hours.

Patients who had never used a computer-assisted insulin self-titration system felt they had enough knowledge to know when their insulin should be adjusted, but still believed that they could use the system to verify their reasoning. Furthermore, patients appreciated that receiving insulin dosing advice at their home could save time and effort. Patients who felt confident in performing self-titration found such a system not useful. Patients who had used PANDIT found the system useful when their glycemic regulation improved. Patients appreciated the ease with which information could be accessed from the system. For some patients, the absence of personal contact with their caregiver was a drawback. While guidelines state that adjustment of basal insulin dose based on fasting plasma glucose values is sufficient [17], both patients who had and those who had not used PANDIT felt that such a system should take more patient data into consideration, such as lifestyle and diet factors.

### Comparison With Prior Work

The first studies that reported on patients’ experiences with regard to insulin therapy focused on patients with T1DM [40]. However, in the United States, it was estimated that in 1999-2000 almost 30% of people with T2DM were treated with insulin [41]. In line with this increase, recent qualitative studies also focused on patients with T2DM, mainly with regard to the resistance to start insulin therapy [23,42], but also with regard to adherence to insulin therapy [29]. However, these studies were large-scale surveys using self-reported questionnaires with a predefined list of possible reasons for insulin discontinuation [23,29,42-45]. The predefined list was restricted mostly to explore patients’ attitudes, either positive or negative, toward insulin therapy and did not cover for example acknowledgment of behavioral change. Furthermore, these studies aimed to collect quantitative information rather than the more “in-depth” and “explorative” qualitative information. The majority of barriers that were found in these studies were also elicited in our study. Important barriers with regard to insulin therapy that were not elicited in our study were: a perceived low efficacy of insulin therapy [23], inadequate explanation of the risks and benefits of insulin therapy by health care provider [42], a physician advising against insulin therapy [45], and the belief that glucose values are under control without insulin therapy [45]. Two previous studies that explored experiences with diabetes self-management of patients with T2DM and also applied in-depth interviews focused on experiences when performing either diet, exercise, or SMBG [24,27]. These studies did not take into regard insulin therapy. Almost all negative experiences referring to SMBG were also elicited in our study. One reason that was not detected in our study causing patients to stop performing SMBG was patients’ inference that health professionals did not consider it important [27]. Nowadays, another important focus of study are barriers to diabetes self-management in general among specific patient groups with low adherence rates [46,47]. So far, we do not know of any study that has investigated patients’ perceptions with regard to insulin self-titration or computer-assisted insulin self-titration, although patients with T2DM increasingly participate in performing insulin titration [48].

### Limitations

A predefined number of 20 patients was included in this study. We did not systematically recruit patients until saturation was achieved, decreasing the likelihood that a complete set of barriers was found. However, the study population consisted of participants with a broad spectrum of age, sex, and HbA1c and was recruited from both general practices and a university hospital. Furthermore, because we had a small number of participants, we were able to perform interviews thoroughly. This increased the likelihood of acquiring a complete set of barriers. Another limitation is the fact that the researchers that conducted the interviews were involved in the development of PANDIT. This may have provoked socially desirable responses by the participants. To increase the reliability of our results, all analyses were separately performed by two researchers. Other than in previous studies that had used in-depth interviews to elicit obstacles to diabetes self-management [24,27], we used theoretical frameworks for the development of the codebook. This provided us the opportunity to position our findings within the existing body of knowledge. Glaser and Strauss acknowledge that no researcher can totally disregard previous literature or theories [49]. However, using a preconceived model may also have introduced bias or contaminated emerging concepts.

### Conclusions

Patients with diabetes on insulin therapy encounter multiple obstacles when performing self-management behavior. Insulin injection itself is painful, a daily recurring burden, and can be embarrassing when performed in the presence of others. Patients experience difficulties with the timing of injections, and with integrating insulin therapy with other therapies and daily activities. Adjustment of insulin dose is a challenge; some patients are ignorant of treatment targets and find it difficult to cope with unpredictable blood glucose levels. In particular, patients have difficulties increasing the insulin dose because they fear hypoglycemia and because they associate higher insulin doses with disease progression. Computer-assisted self-titration can increase patient awareness of treatment targets and increase their confidence in self-adjusting the insulin dose. Nevertheless, some barriers may persist when using computer-assisted insulin self-titration systems, such as reluctance to increase the insulin dose. Furthermore, computer-assisted self-titration could also introduce new barriers. There might be a lack of trust in a computerized insulin dosing advice that, based on guidelines, is primarily based on blood glucose values and does not take account of other patient data, such as lifestyle and diet. Also, for some patients, the absence of personal contact with their caregiver when advice is provided can be a drawback.

### Recommendations for Care Practice

For the design and future implementation of computer-assisted insulin titration systems for patients, we have four recommendations. First, in order to increase the effectiveness of the system in lowering glucose values, the caregiver should verify if the patient is both willing and able to perform SMBG, insulin injections, and to log into a computer-assisted system for insulin dosing advice. Second, to increase acceptance of dosing advice by the system, patients should have the possibility to increase professional involvement through the system, such as by telemedicine functionalities that allow easily accessible consultations with the caregiver. Third, to minimize the burden on a patient’s daily life, the frequency of consulting the system and blood glucose measurements should be decreased once patients have reached near-normal glucose levels. And finally, patients should be motivated to start and continue use of the computer-assisted system by emphasizing its main advantage: achieving lower blood glucose values. This should be emphasized by both the system, for example, by means of a graphical display of blood sugar values, and the caregiver.

### Recommendations for Future Research

We would recommend future research to explore the wishes and needs of patients to interact with their caregiver during the process of insulin titration, in particular, what situations trigger their need to consult the caregiver and what communication modalities would be preferred. Following these potential additional features to the computer-assisted insulin titration system, such as telemedicine functionalities to increase patient-provider interaction, future research should investigate how this would affect patient acceptance.

## Multimedia Appendix 1

Codebook.

## Abbreviations

FPG: fasting plasma glucose
HbA1c: glycated hemoglobin
PANDIT: Patient Assisting Net-based Diabetes Insulin Titration system
SMBG: self-monitoring of blood glucose
T1DM: type 1 diabetes mellitus
T2DM: type 2 diabetes mellitus
