# Supplementary material for: Diabetes Patients' Experiences With the Implementation of Insulin Therapy and Their Perceptions of Computer-Assisted Self-Management Systems for Insulin Therapy
Source: J Med Internet Res. 2014 Oct 23;16(10):e235. doi: 10.2196/jmir.3198 (PMC4259961; doi:10.2196/jmir.3198)
Supplement: Supplementary file 1 [file jmir_v16i10e235_app1.pdf]

Codebook for experiences with and barriers to insulin self-management

In the codebook for experiences with and barriers to insulin self-management, the remarks were first categorized by the medical task (SMBG, insulin injection or insulin titration) and second by the type of experience or barrier (negative/positive experience or reported/induced barrier). Experiences with and barriers to SMBG or insulin injection among patients who already performed the behavior were labeled with constructs from the Theory of Planned Behavior or data-driven constructs. In these cases the 'action stage' construct from the Stages of Change Model is implicit and therefore was not used in the codebook. Otherwise, the constructs 'precontemplation stage' or 'contemplation stage' from the Stages of Change Model were used.

Medical task	Barrier/experience	Construct*	Description	Remark
**SMBG**	Reported barriers	Perceived usefulness (attitude towards behavior)	Participant states that he/she does not perform frequent SMBG because they consider it useless.	For two or three months I performed SMBG in the mornings and afternoons. But the values remained the same. … If I see it [the SMBG result] matches the target value, it's only a confirmation of my values being good. So I have pricked my finger … and it had no use. (participant 4, male, age 40)
		Fit between medical commitment and daily routine	Participant states that his/her busy daily routine (sometimes) withholds them from performing SMBG.	… if I'm busy with something or if I have to go somewhere, it is unpleasant [to perform SMBG]. … It could be that I forget it for a couple of hours; it does not always come at a convenient time. (participant 6, male, age 62)
	Induced barriers	-		
	Negative experiences	Physical impact (attitude towards behavior)	Participant indicates to experience physical discomfort caused by SMBG, for example pain or tenderness in the fingertips or callus formation.	Finger pricking really takes some getting used to, no one likes getting hurt. It gives a feeling of pain. But then again, you know what you need. (participant 2, male, age 54)
				I get sensitive fingertips at a certain moment. I prefer [pricking] three times a day over five times a day. (participant 3, female, age 59)
				In the beginning, when I used to perform SMBG week after week, I got very aching fingers. (participant 10, female, 45)
				I still think finger pricks are a disaster. It just hurts. … I have tried different lancets but all of them hurt. … OK, I have to, I have to, but still I don't like those horrible nasty pricks. (participant 1, female, age 60)
				One week I do it [perform SMBG] every day, then the next week I don't do it. … Because of my fingertips, at a certain moment I can't get through anymore. They have become all calloused. … Sometimes I have to prick twice or three times before a blood drop appears (participant 5, male, age 56)
				Pricking is just unpleasant. … If I'm not careful my fingers will be full of holes. (participant 4, male, age 40)
				It's tough that you have to prick. It would be useful if you could measure [your blood

				glucose] another way, like with a thermometer. (participant 3, female, age 59)
		Perceived cognitive load (perceived behavior control)	Participant states that he/she finds it mentally difficult to perform SMBG in addition to other medical procedures.	I prefer to perform as few medical procedures as possible. I already take medication because my thyroid was removed, that's why I've become overweight and that's how I got diabetes. Therefore I try to limit everything to a minimum. Everybody prefers to be healthy and without needs. (participant 3, female, age 59)
				Also in relation to my use of many medicines … I need a clear structure or I will quickly lose data [SMBG results]. To me it is important because I get older and my memory gets poorer. (participant 9, male, 58)
	Positive experiences	Perceived usefulness (attitude towards behavior)	Participant states that he/she considers frequent SMBG useful.	I find SMBG easy, useful and meaningful. I can immediately see if there is something wrong. (participant 3, female, age 59)
				I used to measure four-six times a day. At a certain point you get acquainted with hypoglycemia and learn to sense a hypoglycemic event coming up. … After a year I knew how much food I had to eat and insulin I had to inject to prevent a hypoglycemic event. (participant 4, male, age 40)
**Insulin injection**	Reported barriers	Perceived cognitive load (perceived behavior control)	Participant states that he/she mentally cannot handle the performance of insulin injection in addition to other medical procedures.	I'm afraid of forgetting to inject my insulin … because I just don't want to think about it. … I have just not accepted the disease and I just want it to disappear. (participant 1, female, age 60)
		Fit between medical commitment and daily routine	Participant states that his/her busy daily routine (sometimes) withholds them from injecting insulin.	Sometimes I just forget [to inject insulin]. Maybe once every five months or so. At such a moment I'm busy doing other things so I just forget. (participant 1, female, age 60)
		Attitude toward behavior	Participant performs insulin injection incorrectly due to his/her negative attitude toward insulin injection.	I always inject after the meal. Actually you should inject before the meal, but I find it intuitively much easier. … First I eat and then I inject the food away. (participant 4, male, age 40)
		Perceived behavioral control	Participant states that he/she finds it difficult to inject insulin correctly.	I administered my insulin before dinner, but we actually ate half an hour later. I was unaware of my low blood sugar level and I just lost it. … As a result, now I wait until I have eaten and then determine how much I will inject. … I could inject a certain amount of insulin before the meal, but if it doesn't add up with what I have eaten, it could be just as well that it was too much or too little insulin. (participant 6, male, age 62)
	Induced barriers	-		
	Negative experiences	Attitude toward behavior	Participant expresses negative feelings toward insulin therapy and insulin injection.	All of a sudden I was confronted with needles. … It was a new situation for me, I didn't know it and I couldn't do it. Suddenly I had to inject insulin. … In the beginning I had to do my best to learn to inject. (participant 2, male, age 54)
				The switch [to a more intense insulin regime] was unpleasant. … The needles were much longer … it was not a pleasant feeling. In the beginning I was pricking in my fingers and

				injecting in my abdomen and things. Initially this was not a nice development. (participant 4, male, age 40)
				You have to consider your food intake, you have to consider administering insulin. Why don't they invent something that requires no injection, [by example] that you get an injection once every three months or so. (participant 7, female, age 46)
		Effect on daily life (attitude towards behavior)	Participant states that insulin injection negatively affects their personal or social life.	You have to take everything into account. In my opinion you are a prisoner. You can eat, but first you need to inject insulin. That's what I think is difficult about it. (participant 5, male, age 56)
				I used to think that I'm going to move to a warm country when I retire. Now I think: would that be possible? Would there be proper health care? … could I get my medication there? That is a limiting factor to move [to a warm country]. It does affect your life for that matter. (participant 3, female, age 59)
				If someone asks: do you want to join us? … I can't, because I did not bring a sandwich … Or if someone proposes at 5 PM to go to the café and have dinner I also can't because I did not bring my injection kit. I always have to carefully consider bringing something or not. (participant 1, female, age 60)
				And also when I go to my family in America, I'm stuck with my insulin. I can't accept that. (participant 7, female, age 46)
				I find injecting insulin difficult in the sense that I have to. It's something I will never get used to and now it becomes a part of my life. … even though you never asked for it. (participant 2, male, age 54)
				I didn't expect diabetes to have that much influence on your private life, or actually on your life in general. It's not only injecting [insulin], but also everything else. ... I also discovered that stress can be of influence. I find it important to take one step back [at work]. (participant 8, male, 61)
				I only drink diet sodas, and I watch what I have for dinner: vegetables with meat, no potatoes, little pasta, as little as possible carbohydrates. … Then I think: what else do I have to let go? It should still remain enjoyable. At a certain moment a doctor said: you should have a walk in the evenings. No way, if I'm at home on the couch in the evenings I don't feel like having a walk and I'm almost 60. (participant 3, female, age 59)
		Physical impact (attitude towards behavior)	Participant indicates to experience physical discomfort caused by insulin injection or insulin, for example swelling or hemorrhages on the abdomen or thighs, or weight gain.	I inject the rapid-acting insulin in my abdomen and I don't like it. I also have bruises on my abdomen and it's more painful and more sensitive. … This wouldn't stop me from injecting, but I do avoid the injection site for some days to give it time to recover. (participant 3, female, age 59)
				[To inject] on the right is easier than on the left. … On the left I also have bruises more often. (participant 3, female, age 59)
				The insulin I used first was stinging. The injection I did not feel, but I did feel the insulin running in. (participant 3, female, age 59)

				I don't like the feeling that I have to inject in my abdomen. (participant 4, male, age 40)
				I find injecting insulin a burden but it does not hurt or something. I use anti-coagulants so I quickly get hemorrhages. (participant 5, male, age 56)
				The once daily insulin causes these swellings at the injection site. They disappear over time. (participant 6, male, age 62)
				I never inject in my legs because I find it terrible. I inject everything in my abdomen. … In the beginning I injected in my thighs but I got swellings and there is no fat on my legs. (participant 5, male, age 56)
				I had much trouble with injecting insulin. My legs got blue. I couldn't accept it, but finally my diabetes nurse taught me how to inject in my abdomen. Injecting in the abdomen is less painful than in the legs. … Nevertheless I can never get used to it. Only fat people feel nothing, but if you are not as fat, you feel it. (participant 7, female, age 46)
				With [insulin A] I gained nine or ten kilos in two years. … it retains fluid. With [insulin B] I did not gain weight. (participant 7, female, age 46)
				First I chose that insulin brand because it causes the least weight gain. But I turned out to be terribly allergic to it and then chose another brand. (participant 3, female, age 59)
		Perceived cognitive load (perceived behavior control)	Participant states that he/she finds it mentally difficult to inject insulin in addition to other medical procedures.	It's very hard to get used to it [insulin therapy], and to learn how to cope with and accept it. … This year was a weird year for me; I have had a mild stroke and lost my visual field. Anticoagulants and report to the thrombosis service. Earlier this year I was at the cardiologist and got more medications. (participant 2, male, age 54)
				I prefer to perform as few medical procedures as possible. I already take medication because my thyroid was removed, that's why I've become overweight and that's how I got diabetes. Therefore I try to limit everything to a minimum. Everybody is preferably healthy and does not need anything. (participant 3, female, age 59)
				I find it difficult that I have to think ahead. It's a heavy burden. … I'm I go on holiday I count everything … because I never know if I'd bring enough. It requires so much preparation that it makes me feel sick. (participant 1, female, age 60)
		Subjective norm	Participant states that individuals important to him/her have negative perceptions of injecting insulin.	If I'm with other people I dislike injecting. In my personal environment my wife is the only one who knows [of my disease]. … I don't feel the necessity to tell others. If I have to inject in public I retreat. I have to say I avoid this kind of situations. I don't often go to family parties and such things. (participant 4, male, age 40)
				I find it difficult to inject insulin when I'm out to dinner. Mostly I go to the restroom. Once I had such a bad experience … People [that saw my insulin pen] thought I was some kind of junkie. I felt really unhappy. (participant 8, male, 61)
				I'm too ashamed to tell everyone that I inject insulin, … many people don't like insulin. I always go to the restroom [to inject my insulin]. And when I'm at a birthday party I wait until I'm home. (participant 7, female, age 46)
	Positive	Perceived	Participant states that he/she	It was when I started using insulin that I saw my blood glucose values were dropping. With

	experiences	usefulness (attitude towards behavior)	considers insulin therapy useful.	tablets my values were too high even though I watched my diet. (participant 3, female, age 59)
**Insulin titration**	Reported barriers	Timeline	Participant thinks that increasing the insulin dose means he/she is getting sicker	… they said increase [your insulin dose] to 18 [insulin units], increase to 24. Then I thought: it's getting worse. Am I getting sicker? … I didn't know this was normal in the initial phase. … It takes some time before you realize how it works. (participant 2, male, age 54)
		Consequences	Participant is afraid of short time consequences of increasing the insulin dose	[I used to have difficulties getting the right [blood glucose] value. There were peaks and troughs and you have to prevent that.] … I was afraid to inject too much [insulin] and consequently get a hypoglycemic event; that is very unpleasant. (participant 6, male, age 62)
		Controllability	Participant states that he/she finds it difficult to titrate his/her insulin dose, because he/she has no control over the diabetes treatment, resulting in insufficient or incorrect insulin titration.	Adjusting the insulin dose is difficult, if I have the flu my [blood glucose] values go straight up to 18 or 19 [mmol/L]. In such cases I inject an additional 6 insulin units or something. (participant 5, male, age 56)
				I find it very hard to adjust my insulin dose because it's so difficult to keep something stable. There are so many external factors … I'm just very busy at work or sometimes I have a lousy night's sleep because your neighbor is giving a party or just because I'm not feeling well. It has a direct impact. I always ask myself: what could be the cause [of my deviating blood glucose value] but I don't always know. … Now I realize that I just have so little influence and I think: I try so hard and feel I'm doing well, but then still my sugars are high. (participant 3, female, age 59)
				I try to listen to my body to see whether my blood sugar is low or high. But maybe I misjudge, because my eyes could also be red due to high blood pressure. (participant 2, male, age 54)
				I never notice if my blood sugars are high. … because I can't sense it, I always prick [measure my blood glucose level]. This morning I had a good sleep and I thought my [blood glucose] levels were great, but they were at 9.6 [mmol/L] and that's very high. (participant 3, female, age 59)
				I used to have difficulties getting the right [blood glucose] value. There were peaks and troughs and you have to prevent that. (participant 6, male, age 62)
		Perceived behavioral control	Participant states that he/she finds it difficult to titrate his/her insulin dose.	I'm not increasing [my insulin dose] until they [diabetes nurses] say what to do. … It's dangerous, … I don't know if I inject more [insulin] it will give problems, with my other medications or something else. (participant 7, female, age 46)
				I wouldn't increase my insulin dose if I thought my blood sugar were too high because it fluctuated so much. I don't understand how that was possible. I don't like eating candy, I only drink diet beverages and no alcohol, I don't smoke… (participant 7, female, age 46)
		Controllability / fit between	Participant states that his/her busy daily routine	I have had trouble getting it [the blood glucose levels] stable. I used to work irregularly with irregular working hours. … If I have to wake up at 4AM for three days in a row, and

		medical commitment and daily routine	(sometimes) withholds them from correct insulin titration.	after three days I start at 2PM, then I sleep late and measure my blood sugars later. .... Now I work from 8AM till 6PM so I can measure my blood sugar at the same time and can inject at the same time. Because the irregularities just didn't work with me. (participant 8, male, 61)
	Induced barriers	consequences	Participant titrates his/her insulin dose incorrectly due to incorrect information on the treatment objectives.	I always fear that my [blood glucose] value shouldn't fall below 5 point something [mmol/L]. (participant 2, male, age 54) … is deze zin correct?
				It's best if my blood sugar in the mornings is between 5 and 7 [mmol/L]. This morning it was 4.4 [mmol/L] so I injected 2 insulin units less. I injected 20 units instead of 22. (participant 5, male, age 56)
				For me between 5.5 and 6.5 [mmol/L] is normal. If it's lower I think: this is getting too dangerous, so I decrease my insulin [dose]. Then I see how it's going and most of the time it's fine. (participant 3, female, age 59)
				If my blood glucose value is near 10 or 10.2 [mmol/L] I'm not very alarmed. … If it would be near 15 [mmol/L] I could call [my diabetes nurse]. … Until 10 point something it does not bother me. (participant 2, male, age 54)
		Controllability / attitude towards behavior	Participant titrates his/her insulin dose inadequately due to the perception that adjusting the insulin dose does not provide control over his/her disease.	If you inject more insulin if your blood sugar is too high, you end up in a situation where you're dependent on [exogenous] insulin. Say you have a steady lifestyle and a fixed insulin dose. … If your [blood glucose] values decrease it could mean that the Islets of Langerhans function better. If you constantly switch insulin doses, you can't see if you're doing better. (participant 9, male, 58)
				I use those [SMBG] results for making adjustments to my lifestyle. … I'd rather not touch my insulin dose. Then the transparency of those [insulin] units will decrease … If increasing the insulin dose is a consequence of an incorrect lifestyle, it means that you shouldn't look for the solution in the insulin dose, but in other things. … I think my insulin dose can be lowered by 30-40%, given the fact that I'm exercising again. Now I use 38 units, later I might need 20 and in the mornings 4. And maybe in the end I use none at all, and that's my aim. (participant 9, male, 58)
	Negative experiences	Attitude toward behavior	Participant expresses negative feelings toward insulin titration.	My values with that insulin brand were 8-9 [mmol/L]. With the other insulin it was higher, 11 or 12 [mmol/L]. Then my diabetes nurse said we're going to increase your insulin dose and I thought: oh God. (participant 7, female, age 46)
				My blood glucose is regularly under 5 [mmol/L] in the morning. … then I don't worry. But I do when my evenings are around 2 [mmol/L]. Say something would happen, what is there you can do? … I wake up transpiring terribly and don't feel well. I worry about that … if you go to sleep, what could happen? (participant 8, male, 61)
				I don't find it more difficult to increase [my insulin dose] that to decrease, but the only thing is that you can inject till 38 [insulin units] in your leg, but if you use 40 units you have to divide it in two times 20 units or you get hemorrhages. That's the only thing that's inconvenient, I have you twist the needle on twice and inject twice. (participant 3, female,

				age 59)
		Controllability	Participant states that he/she finds it difficult to titrate his/her insulin dose, due to which he/she feels that he/she has no control over the diabetes treatment.	We changed the insulin because it didn’t have the right effect. With the new insulin I inject twice a day, but my [blood] sugar still didn’t decline. … In the mornings my fasting [blood glucose] value was 10 or 11 [mmol/L]. In the evening before going to bed it was 18, sometimes 20 [mmol/L]. And that is very high. (participant 7, female, age 46)
				I had already increased two insulin units, but it did not have effect. Then last night I increased it again from 38 to 40 units, and [this morning] my blood glucose was still at 9.6 [mmol/L]. … How unfair. (participant 3, female, age 59)
				I proposed to start with another brand of insulin because my first brand peaked at nights and didn’t work during the day. (participant 3, female, age 59)
				With my insulin [brand] I used to feel in the mornings that I had to sit down or I’d lose it. At those moments I sit down and have some sweets. (participant 10, female, 45)
				It didn’t go well with my insulin. … Often when I didn’t eat anything … my blood glucose value was high. And another time when I had a cookie my value was fine. I was confused, there is no logic in it. I couldn’t understand it, because I tried my best; no diet code, nothing, but then my value went up. (participant 7, female, age 46)
				Sometimes when I come from the gym my [blood glucose] value is higher compared to when I wake up and haven’t eaten anything. But it’s because of the adrenalin. Also sometimes when my fasting value is nicely at 4.5 [mmol/L] and I go to the lab to test my blood it turns out to be 7.5 [mmol/L]. Then I’m confused, it probably is because I have cycled. (participant 3, female, age 59)
				In the initial phase I had trouble adjusting my insulin dose to get my blood sugar levels stable. In that area I was supported by the diabetes nurse. … At a certain moment I understood the effect of certain food. (participant 4, male, age 40)
				I feel I’m not handling my insulin adjustments adequately. Sometimes I wonder if I’m waiting too long and I don’t feel like increasing [the insulin dose]. Sometimes I really feel like I’m messing around, like: let’s increase [my insulin dose] again. Am I doing better? No. And then suddenly my [blood glucose] values are good again and I think: how is that? I just don’t understand.” (participant 3, female, age 59)
	Positive experiences	Controllability	Participant states he/she feels control over their diabetes treatment.	If I had dinner with friend and had sweetness as dessert you’d think my [blood sugar] value would be high, but because I walked from the subway home my value is fine. … Physical activity has a lot of influence. (participant 3, female, age 59)
				Sometimes I make long trips to Vietnam, and it goes very well, because my values are much lower than in the Netherlands. My diabetes nurse that has to do with the warmth. Moreover I eat less and exercise more so I perform SMBG more often. Mostly I decrease my insulin dose. … The other day I was on vacation to Istanbul with friends and it went perfectly. I was more relaxed and exercised more. (participant 3, female, age 59)
				Doctor S. came and said that surgery would help me get my [high blood glucose] values

				down. And he was right. I've had a gastric bypass and my sugar went down! … Now I take one pill a day … and 28 insulin units in the mornings and 30 units in the evenings. … I feel great, oh so great. … After the surgery I had a lot of pain for four weeks and was upset because I could only eat porridge and apple sauce and my abdomen was blue. But now it's going well and I got fit. (participant 7, female, age 46)
**Insulin initiation/ insulin intensification**	Reported barriers	Contemplation stage	Participant states he/she is not sure of wanting to initiate insulin therapy or intensify insulin regime.	I started out with tablets. … Then they said 'you're going to start with insulin'. But I was against it, because I feared injections. … I started [injecting] in my legs. … It was so painful. It took me one year to get used to [injecting] insulin, because of the lifetime commitment. Why not pills? (participant 7, female, age 46)
				I just can't stand the injection. If it would be once a day it would be fine. But I had to inject twice a day. … Two times is really the maximum for me. They proposed to me to inject three times, but I said no. I don't want that. I couldn't do it. (participant 7, female, age 46)
				My diabetes nurse said: if you see your blood sugar increasing, inject some more insulin. My sugar [levels] were a bit high, but I didn't want to inject three times a day. … because if I do, they will say I have to keep up the three times daily injection and I wouldn't want that. … And then from three times to four times. (participant 7, female, age 46)
	Negative experiences	Contemplation stage/ attitude toward behavior	Participant expresses fears of disease progression when initiating insulin therapy or intensifying insulin regime.	It was more an emotional step to switch from tablets to insulin. You have to do it lifelong and you will never get rid of it. It felt like this was the next stage of being sick … I felt more of a patient. (participant 3, female, age 59)
				Maybe a time will come where I have to use insulin thrice-daily. That's not something I look forward to. (participant 3, female, age 59)

* Experiences with and barriers to SMBG or insulin injection among patients who already performed the behavior were labeled with constructs from the Theory of Planned Behavior or data-driven constructs. In these cases the 'action stage' construct from the Stages of Change Model is implicit and therefore was not used in the codebook. Otherwise, the constructs 'precontemplation stage' or 'contemplation stage' from the Stages of Change Model were used. For the remarks specifically about insulin titration, where possible, an additional construct from the Self-Regulatory Theory was used.

Codebook for perceptions of computer-assisted insulin self-titration

Patient group	Construct/codes	Description	Remark
**Patients who have never used a computer-assisted insulin self-titration support system**	Trust	Degree to which patients think they can trust the dosing advice	I think it's a good idea [to develop an online system that gives daily insulin dosing advice]. But … I listen to my body because I have been in the situation before where I put my trust in someone, but then there is a shortage of knowledge. It's OK, no one is perfect, we all have to learn. But it makes you realize you have to be careful with what you're presented with. You shouldn't blindly rely on everything. (participant 4, male, age 40)
	Dependibility	Degree to which patients think they can depend on the system	When it [a computer system] crashes, you lose everything. I have a problem with that. You're totally dependent. (participant 4, male, age 40)
	Completeness	Degree to which patients think the system incorporates or provides all necessary information	I think it [computer-assisted insulin self-titration] might not be realizable, because the system will not know your exercise rhythm. If I go fishing one day, then I know what insulin dose I'm going to administer. But if I suddenly give up fishing and go for a 90 minute run, I have already entered my data, and they [caregivers] will not know that I'm exercising. … I think you should always listen to your body. Either way, I wouldn't say no to it [using such a system], I would be open for trying it out. (participant 5, male, age 56)
			On the one hand I think it would be meaningful if you receive insulin dosing advice. On the other hand, based on what my diabetes nurse said I think there are many other factors [that play a role in the determination of an insulin dose] than just the [fasting] blood glucose values. … I think it could be dangerous if you just rely on such an advice. For example if you'd happen to take antibiotics, causing it [the fasting blood glucose level] to be increased, or when you have a fever. You shouldn't solely rely on it, it should be for support only. (participant 3, female, age 59)
			It would be a good idea [to develop a system for insulin self-titration]. It can take into account several things, such as how much you have eaten and how you feel about it. (participant 4, male, age 40)
	Efficiency	Degree to which patients think the system is efficient	It [the use of a device that can help me adjust my insulin dose] could save me time. Sometimes you have to wait here [in the hospital] for quite a while. … Other times there are telephone appointments but that also requires waiting if the line is busy. The use of such a system would save both parties time. If it's easy and possible [to titrate the insulin dose] without speaking to the person I would like that, yes. (participant 2, male, age 54)
			I think it [such a system] would be very helpful, because it also provides certainty for someone. When you have high [blood glucose] values … it's difficult to get a diabetes nurse on the phone. They are only available certain hours a day. (participant 1, female, age 60)

			That way I wouldn't have to come [to the hospital] and still get new [insulin dosing] advice. I would like to use it [such a system]. (participant 6, male, age 62)
			I would find it [using such a system] useful. I wouldn't have to visit every time and meanwhile I use the Internet to get in contact [with the diabetes nurse]. (participant 8, male, 61)
			We [me and my diabetes nurse] agreed to see each other once a month and we quickly learned that this [sending e-mails in the meantime] took far less time. Currently I'm going to three different hospitals and there are also other things you have to do in life. (participant 9, male, 58)
			You could connect it [such a system] to an electronic patient record. That way the diabetes nurses, researchers and the general practitioner or specialist could work together. (participant 9, male, 58)
	Perceived usefulness	Degree to which patients think the system is useful	I don't see a surplus value in using a device to download your fasting blood glucose values. But if you'd get useful insulin dosing advice in return, it would be a different story. (participant 3, female, age 59)
			I would find it [an internet-based system providing insulin dosing advice] useful. You could use it to discuss certain actions. You wouldn't have to visit the clinic every time … Such an advice would be a sort of confirmation. In the beginning I used to do this with my general practitioner, by sending [e-mailing] him my [blood glucose] values every five days. (participant 8, male, 61)
			I think that such a system would give more certainty before adjusting my insulin dose. (participant 10, female, 45)
			I'm not sure if it's useful to use such a system daily if you already know what to do. I come to the clinic once every three or four months. In between I don't call because we have discussed how much insulin I should use. … I'm not sure if it's useful if I perform SMBG but then first check what the computer says [what insulin dosing advice is given]. (participant 6, male, age 62)
			I think you can just as well get in touch with your diabetes nurses by telephone. … I have done that several times. (participant 6, male, age 62)
			Why would I want to regulate my [basal] insulin? … I don't think it's useful for me … perhaps [it would be useful for] people who have difficulties with their diabetes and don't have it for as long as I do. (participant 1, female, age 60)
	Preferences	Whether patients prefer this method of receiving dosing advice.	I normally don't give out my e-mail. I find e-mailing impersonal, I prefer to use the telephone. (participant 5, male, age 56)
**Patients who have used PANDIT in a pilot study**	Completeness	Degree to which the system incorporates or provides all necessary information	Currently it's [the determination of new insulin doses] only focused on the glucose values, but of course other influences are nutrition and lifestyle. Would it be useful to extend the system with information on the lifestyle and food intake? (participant 18, male, 64)

			Because of my irregular working hours I'm trying out different things [in the online diary]. For instance if I'm on a night shift I don't sleep and only drink water. In the afternoon when I get out of bed I measure my fasting blood glucose. After that I have breakfast, so I turn my days around. So [in my diary] I just write: 'measured during the day because of night shift'. (participant 14, male, 47)
			Normally my [blood glucose] values are very low, but then once I had a major outlier. The night before I had a party and had quite some snacks. Then it's logical that my value is very high. I entered that in the comment box. … so if the nurse notices it she'll understand why it's [the value] is so high. (participant 11, female, age 47)
	Format	Perception of how well the information is presented	It might be useful if those difficult words like 'hypo-something' [hypoglycemia] and such are replace with just 'hypo'. (participant 18, male, 64)
	Flexibility	Way the system adapts to changing demands of the patient	In response to the question I sent to my caregiver [through the e-mail functionality of PANDIT] I received adequate reply, which was very nice. (participant 18, male, 64)
			I have had several contact moments [with my caregiver]. Normally I injected in my abdomen, but then I was being advised to inject in my leg, and I asked a couple of questions through the system. (participant 11, female, age 47)
			I was wondering if this was a good place to add comments. Because … if I have a pain attack or the flu everything gets disrupted. If I let them [the caregivers] know how the high or low [blood glucose] value occurred, they can intervene if necessary. (participant 18, male, 64)
			I fill in my blood sugar values in the diary and add comments, but I don't get feedback on those. Why else would you be able to write a comment? Look, if my comment would be 'I have eaten something so I might deviate a little' she wouldn't have to comment on it. But when I felt so miserable I would've liked some feedback. (participant 12, female, 53)
	Accessibility	Ease with which information can be accessed from the system	I'm not sure if there's a possibility to look back at things. For instance if I've had contact the caregiver, can I see what I have written earlier? (participant 18, male, 64)
			Perhaps it is easy to handle it [the filling in of the diary] on your mobile phone. … When I'm on holiday I do have an internet connection on my mobile phone and it would be ideal if I could log on to PANDIT through my phone. (participant 14, male, 47)
			I fill in my values and remarks and after three days the system says: you have pricked enough, you will receive a new [insulin dosing] advice. Everything went well. (participant 12, female, 53)
			I just quickly update the diary and I don't worry about anything else. (participant 13, female, 62)
			If I have measured my fasting [blood glucose] values, I type it in, enter whether I was on night shift and then I'm finished. Then I continue the next day. (participant 14, male, 47)
			I haven't used any functionalities. I nearly filled anything in and automatically got response when I had to increase [the insulin dose]. (participant 15, male, 50)

	Perceived usefulness		I'm used to do it [titrate the insulin dose] all by myself. Once every three months I go to see my nurse and she says I'm doing it right or wrong. I already use a diary and decide whether I'm going to inject more or less [insulin]. (participant 17, male, 59)
			I think PANDIT is especially meant to be used by people whose blood glucose values fluctuate a lot. I'm actually always between 4.8 and 6.5 [mmol/L] so it would always give the same advice. (participant 17, male, 59)
			Now that I measure my fasting blood glucose values [and enter my fasting blood glucose values in the system] my daily sugar value is around 6 whereas it used to be over 10 [mmol/L]! I'm very happy with the system. … I only feel better. Now my insulin dose is substantially increased. I think I started on 24 and now I'm on 52 [insulin units] so for me it has been very good advice. (participant 11, female, age 47)
	Attitude toward behavior		Normally I only pricked [perform SMBG] if I wasn't feeling well, but now I participated in the pilot study to see how it is. I find pricking daily a bit disturbing. Normally I prick twice or three times a month. (participant 14, male, 47)
	Missing functionalities		I preferably call or talk face-to-face rather than I send an e-mail. I like looking at people to see how it is said … and what they mean. … With e-mail you have to wait for a reply, but if you call or ask something face-to-face you get immediate response. … I prefer some sort of human touch. (participant 15, male, 50)
			I miss the personal contact with the diabetes nurse. … In August I started with insulin and every two weeks I called my diabetes nurse. I had personal contact by telephone, and even came to the hospital a couple of times, and now things go through e-mail. I find contact by telephone more enjoyable than by computer. (participant 16, female, 68)
